# The Sir Ludwig Guttmann lecture 2023: psychosocial factors and adjustment dynamics after spinal cord injury

**DOI:** 10.1038/s41393-025-01060-6

**Published:** 2025-01-17

**Authors:** Ashley Craig

**Affiliations:** Rehabilitation Studies, Faculty of Medicine and Health, The University of Sydney, The Kolling Institute, Northern Sydney Local Health District, St Leonards, NSW Australia

**Keywords:** Risk factors, Signs and symptoms, Health care

## Abstract

**Study design:**

Narrative review

**Objectives:**

Sir Ludwig Guttmann realised spinal cord injury (SCI) rehabilitation should incorporate more than a biomedical approach if SCI patients were to adjust to their injury and achieve productive social re-integration. He introduced components into rehabilitation he believed would assist his patients build physical strength as well as psychological resilience that would help them re-engage with their communities. We pay tribute to Sir Ludwig by presenting research that has focussed on psychosocial factors that contribute to adjustment dynamics after SCI.

**Setting:**

Not applicable.

**Methods:**

Five factors with a psychosocial source will be examined, featured in my research, namely psychological distress, cognitive impairment, pain catastrophizing, sleep disorder and fatigue. A multifactorial model of adjustment will be examined.

**Results:**

Evidence shows these factors can be significant barriers to adjustment and reciprocally related to self-efficacy and life decisions. A theoretical rehabilitation framework/model is presented called the SCI Adjustment Model (SCIAM), that explains the process of adjustment dynamics. It describes how multifactorial factors contribute to adjustment in a non-linear process over time.

**Conclusions:**

Key clinical messages include: (i) adjustment dynamics will be enhanced if viewed through the lens of a multifactorial model that clarifies how multiple psychosocial factors can combine and act as barriers or facilitators to adjustment; (ii) judiciously using this information, assess and then strategize to reduce the influence of barriers or strengthen facilitators during SCI rehabilitation and beyond, and (iii) integrate psychosocial guidelines and a person-centred approach into SCI rehabilitation to achieve treatment goals.

## Introduction

The 2023 Sir Ludwig Guttmann Lecture was delivered at the International Spinal Cord Society conference in Edinburgh, Scotland. Sir Ludwig Guttmann’s initiatives in spinal cord injury (SCI) rehabilitation were groundbreaking [1]. He established the SCI Unit in the mid-1940s in Stoke Mandeville, Buckinghamshire, England, now the National Spinal Injuries Centre, United Kingdom. By the late 1940–60s, based on his clinical experience and the challenges of injuries resulting from the Second World War, he began to view SCI rehabilitation as more than a biomedical problem. Problems of adjusting back into society were common as his patients were largely unprepared for community living after being treated in hospital, having a serious impact on their recovery [1]. He championed this position by incorporating into SCI rehabilitation in Stoke Mandeville activities such as physical activity and sport [1]. He reasoned this would increase physical and psychosocial strength, and consequently improve social re-integration [1].

SCI rehabilitation and adjustment is a complex process with the pathway to recovery requiring specialist expertise best provided in a SCI Unit staffed by a multidisciplinary team (MDT). SCI rehabilitation begins in the intensive care and acute care settings where the individual needs of each person are addressed [2]. After determining the level of motor injury and an assessment using the American Spinal Injury Association Impairment Scale grade (AIS), neurologic recovery outcomes can be established and functional goals planned [2]. The World Health Organization (WHO) also considers SCI rehabilitation a goal-oriented process intended to optimize the healing of remaining function, and which results in the maximum possible physical, psychological, social and economic independence [3]. This involves coordinated teamwork of the MDT within a person-centred care (PCC) context, in conjunction with social, educational and vocational services [4, 5]. PCC is an inclusive approach characterized by individualized care, based on the needs of each person that encourages them to play a role in their care [4, 5]. See Table 1 for essential PCC criteria. SCI rehabilitation is a process that involves not only physical/medical recovery, but crucially, it also requires social, psychological and cognitive recovery leading to optimal social reintegration of the person into the community, including return to employment if possible [4–6].

**Table 1:** Essential person-centred care criteria that should be employed in SCI rehabilitation [4, 5].

1. The person with SCI has their own set of unique needs, preferences, values, emotions, beliefs, worries and expectations
2. The person with SCI should be involved with health professionals in decision making and treatment planning relevant to their rehabilitation
3. Genuine caring and reciprocal relationships are developed between the person with SCI and the MDT
4. Open communications are cultivated between the person with SCI and the MDT health professional, composed of appropriate socially skilled verbal and non-verbal behaviour, using open-ended questions, simplifying information, and making eye contact
5. The MDT health professional is experienced and expert in SCI rehabilitation, committed to evidence-based practice, understands basic psychological skills, and maintains a tolerant, respectful, compassionate commitment to the person with SCI
6. Teamwork within and between disciplines/healthcare providers in the MDT is fundamental, including up-skilling teams through training and educational programs

*MDT* multi-disciplinary team.

## Methods

Using a narrative review approach, five factors, which have been a dominant feature in my SCI research, will be examined critically in the literature for evidence that suggests they can act as barriers or facilitators to adjustment after SCI. These factors include psychological distress, cognitive impairment, pain catastrophizing, sleep disorder and fatigue. Further, adjustment dynamics will be viewed through a multifactorial non-linear model that explains the contribution to adjustment over time of moderators and psychosocial mediators. The importance of PCC, peer support and psychosocial guidelines will also be explored within the context of SCI rehabilitation dynamics.

## Results

### Adjustment after SCI

The majority of adults with SCI, perhaps as high as 60%, cope and adjust well over time after their injury and its associated impairment, even though they face substantial daily challenges [6]. The reality is that most people with SCI will experience difficulties that challenge their resilience [7–9]. Difficulties include debilitating conditions such as respiratory infection, bowel and bladder infection, chronic pain, pressure injuries, sexual dysfunction, and cardiovascular dysfunction such as autonomic dysreflexia [7, 8]. Due to its life-changing nature, SCI will influence every aspect of a person’s life, including psychosocial aspects, such as interpersonal relationships, where partners or family members of an adult with SCI are vulnerable to relationship conflicts and breakdown in the first 2-years post injury, with many experiencing elevated anxiety and depressive mood [10]. The following discussion will assist in better understanding the influence of psychosocial factors that can act as barriers or facilitators to adjustment [5]. Whatever one’s view of this area, I believe if psychosocial barriers are left unaddressed, then SCI management and rehabilitation outcomes will become more complex and costly, ultimately leading to decreased QoL and unacceptable restrictions in social re-integration [5, 6].

### Definition of psychosocial

The term “psychosocial” has had a variety of definitions in the health professions [11]. Here, a brief definition is offered based on a more comprehensive definition [11]. The concept of “psychosocial” primarily involves a person’s attitudes and perceptions about their psychological, physical, social, environmental, and spiritual status. The definition assumes that a person’s perceptions influence their behaviour related to their social access and mobility, their interpersonal dynamics with family and social networks, their environment, and wider society structures like education, culture, religion, and legal systems [11]. A definition of “psychosocial” also incorporates the idea that some factors (e.g. age, disability, physical health, material resources, and social support at the individual or social level) may improve or lower the psychosocial health of an individual [11]. Finally, “psychosocial” involves the dimension of time, in which its influence on health will vary over time, dependent on how these factors influence the person, their context and how factors interact with each other. For example, increasing age and presence of secondary conditions like pain, will more than likely be associated with decreasing cognitive capacity [5]. This definition of “psychosocial” will be applied in the ensuing discussion on factors that impact adjustment dynamics and will be applied when discussing a multifactorial model of adjustment dynamics.

### Psychosocial impacts after SCI

There are many psychosocial factors that affect adjustment dynamics after SCI [5, 6, 12]. Research has consistently shown that adjustment after SCI is not only influenced by level and completeness of the injury and presence of physical co-morbidities, but also by psychosocial elements [5, 6, 9, 12]. For example, in a study examining resilience after SCI, psychosocial factors like self-efficacy were found to contribute significantly to resilience, whereas injury and demographic factors did not contribute significantly 12-months post SCI [13]. A recent narrative review focused on the adverse impact of psychosocial factors after SCI, focussing on relationships and family, finances and employment, the person’s living environment, community reintegration and sexual health, using the framework of the biopsychosocial model [12]. Readers are directed to this interesting paper for more detail [12]. In this paper, I examine five additional psychosocial factors which can act as significant barriers to adjustment. Much of this evidence has originated from my research. These factors include psychological distress, cognitive impairment, pain catastrophizing, sleep disorder and fatigue.

#### Psychological distress

The majority of adults who sustain a SCI, perhaps as high as 60–70%, will cope and adjust suitably, despite the challenges associated with this severe injury and lifelong disability [6, 13]. Nonetheless, most, if not all people with a SCI will initially experience grief and significant sadness as a consequence of the injury, which, though distressing, is considered an adaptive reaction to SCI [14]. Worryingly, evidence suggests that up to 40% are at risk of experiencing difficulties with sub-clinical or clinically elevated psychological distress and mental health disorder [5, 6, 9, 15, 16]. Results from a meta-analysis confirmed that psychological distress is clinically elevated after a SCI compared to able-bodied controls [15], with data indicating that distress had only begun to reduce 10 years post SCI [15]. A further meta-analysis investigated the prevalence of depression after SCI and concluded that around 26% of adults with SCI met a diagnosis of probable depression depending on the time post-injury [16]. Rates of post-traumatic stress disorder and anxiety disorders in adults with SCI are also higher than community rates [9]. Research that employed gold standard psychiatric interviews for a diagnosis and which followed adults with SCI prospectively from the acute stage of the injury to 12-months post injury, concluded that up 30% met psychiatric criteria of any psychological disorder [9]. Psychosocial factors significantly contributed to the risk of elevated levels of psychological distress 12-months after the SCI, including lack of social support, no care partner, catastrophizing thinking, and chronic pain [9]. Premorbid psychological treatment was a very strong identifiable risk, with the odds of developing psychological distress 12-months post SCI almost 24 times greater than those for someone with no premorbid history of psychological distress [9]. The same study established that psychological co-morbidities are also high after SCI [9]. Around 10% of participants met psychiatric criteria for two psychological disorders and 3% met criteria for three psychological disorders [9]. Major depressive disorder and substance use disorder was the most common co-morbidity, followed by major depressive disorder and suicidality. About 4% had a co-morbid PTSD and /or generalized anxiety disorder with depression [9]. Rates of PTSD with co-morbid pain is higher in veterans who sustained a SCI while serving in the military [17]. Evidence suggests that most if not all psychosocial challenges will act to increase or decrease psychological distress depending on the person and their circumstances [5, 6, 12].

#### Cognitive impairment

Cognitive impairment is a prevalent secondary health condition after SCI [18, 19]. It has been estimated that 12-months after the injury, the odds of an adult with SCI having cognitive impairment was about 18 times greater than that for someone without SCI [19]. Prospective research found that executive function, attention, perceptual (visuospatial), memory (learning), and language domains were all significantly reduced compared to an able-bodied control group [19]. However, a recent network meta-analysis concluded that deficits in cognitive function after SCI mostly occurred in the attention and executive function domains, while language and perceptual domains were least affected [18]. The presence of cognitive impairment in adults assessed in the acute and rehabilitation phase after SCI can have increased risk of adverse impacts on their mental health after discharge from hospital, when transitioning into the community, a time when personal resources will be challenged [19]. Without the comprehensive support provided during inpatient SCI care, those people with cognitive impairment will be more likely to believe their disability is beyond their control and feel helpless about their disability, making them vulnerable to poor self-management and associated secondary health conditions like infection, depression and clinically elevated anxiety [5].

Many factors can contribute to the development of mild cognitive impairment after SCI [19]. Traumatic brain injury (TBI) if comorbid with the SCI, can be associated with cognitive impairment [19]. Other factors that can contribute to cognitive impairment after a SCI include pre-morbid brain trauma and learning difficulties, fatigue, sleep disorder, chronic pain, elevated anxiety and depression, polypharmacy, substance abuse, older age and post-traumatic inflammation [19]. Treatments investigated for improving cognitive impairment after SCI are in their infancy, so this is a challenge for future work [20].

#### Pain catastrophizing

Chronic pain after SCI is commonly associated with other comorbid psychosocial conditions like fatigue, depression and functional limitations [5], and is also commonly associated with cognitive biases like pain catastrophizing and low self-efficacy in people with SCI [5, 21]. Pain catastrophizing involves negative cognitive appraisals including feeling helpless and overwhelmed, intrusive and repetitive negative thinking called rumination, and magnifying their fears, thinking how horrible and disastrous their life is [5, 21]. Pain catastrophizing can be a serious barrier to adjustment after SCI [5] and it will interact with other psychosocial factors to worsen outcomes, like low self-efficacy, the social environment, sleep disorder, excessive daytime sleepiness, fatigue and psychological distress [5, 21]. In prospective research following adults with SCI from rehabilitation and into the community 12-months after the injury, pain catastrophizing increased substantially in those with clinically elevated mood states during rehabilitation, while in those with normal mood, pain catastrophizing did not alter significantly over this time period.

This finding supports a multifactorial model that predicts that depressive mood will act as a moderator on catastrophizing [21]. This is clinically relevant because adults with SCI who are depressive have a higher risk of catastrophizing about their pain and our data also showed that these people will have higher levels of pain intensity [21]. This prospective data also indicated that the social environment into which the adults with SCI were discharged can exert an adverse impact, resulting in increased pain intensity and catastrophizing [21]. When adults with SCI are discharged into the community following their intensive rehabilitation, they of course will receive less professional support and assistance from the MDT, and consequently, their resources required to adjust will be challenged and they will often feel stressed and overwhelmed. It is crucial this barrier to the adjustment process be addressed by investigating effective counter-cognitive management strategies for strengthening self-appraisals and coping skills [5, 21].

#### Sleep disorder

Sleep disturbance such as obstructive sleep apnea can be a significant barrier to adjustment after SCI [5, 22]. Poor sleep quality has been strongly associated with other psychosocial problems like negative emotional wellbeing and low vitality, higher rates of unemployment, and greater problems with participation in people with SCI [22]. A related barrier is excessive daytime sleepiness, described as a susceptibility to falling asleep during the day [23]. Sleep disorder and excessive daytime sleepiness will diminish QoL and social participation, especially in those with cervical and thoracic lesions and sensorimotor complete injuries [5, 23]. Chronic pain can significantly disrupt sleep quality, and conversely, poor sleep can exacerbate levels of pain [23]. It has been argued that inadequate sleep and accompanying sleepiness will reduce top-down cortical/cognitive resources (e.g. attention), leading to decreased capacity to manage pain, and increasing risks of psychological distress [21, 23]. I recently examined the influence of pain catastrophizing on the ability of adults with SCI to stay awake during a behavioural test of daytime sleepiness called the Oxford Sleep Resistance Test [23]. If high pain catastrophizing was present, sleepiness was severely affected, with 70% of participants with SCI falling asleep, compared to 33% falling asleep for those with minimal catastrophizing [23]. Findings suggest significant sleep benefits may occur in adults with SCI by treating cognitive biases like catastrophizing, as well as addressing associated factors like fatigue, pain interference, low mood, and so on. This study again demonstrates how psychosocial factors can interact to créate even more problematic barriers to adjustment [23].

#### Fatigue

Chronic fatigue is another debilitating problem after SCI [5, 24]. It is a multifactorial condition that can be defined as excessive chronic tiredness involving feelings of physical and mental exhaustion and negative emotions, such as anxiety and poor mood [5, 24]. Major problems associated with fatigue can include increased chances of errors when performing tasks, reduced motivation, circadian rhythm disruption, and increased risk of anxiety and confusion [24]. In a controlled experimental condition requiring mental concentration over a period of 2–3 h, adults with SCI and able-bodied controls were compared for the extent that they experienced significant tiredness [24]. While the able-bodied participants experienced some tiredness, they did not reach extreme levels of fatigue, while 56% of the SCI participants experienced serious fatigue [24]. Psychosocial factors found to contribute to the development of fatigue consisted of poor self-efficacy and depressive mood [24]. Many people who sustain a SCI are of working age and employed at the time of their injury, so re-engagement in employment is an important goal of SCI rehabilitation and functional recovery. Re-employment can be associated with improved functioning, adjustment, self-esteem, wellbeing, financial independence and social integration, leading to higher QoL [24]. However, rates of employment remain low after SCI and psychosocial factors like fatigue interacting with other psychosocial barriers, will have a major impact on their ability to return to work [24].

### A unified SCI rehabilitation management framework

I have endeavoured to illustrate how the above five psychosocial factors impact adjustment after SCI, and how they can interact to create barriers to positive adjustment. It is important now to consider a framework that incorporates such impacts and interactions. The Biopsychosocial Model of care provides a unified rehabilitation management framework for SCI rehabilitation, especially when delivered within the context of a PCC approach [4, 5]. Such a model views a person’s rehabilitation, health and well-being as the outcome of interacting physical, psychological, and social domains, where psychosocial domains of intervention are equally important as the physiological and medical domains [4, 5]. Each domain is necessary and is expected to play a critical role in helping the person to adjust and thrive [5, 13]. It is my belief that SCI rehabilitation delivered in such an atmosphere will optimize and enhance adjustment and resilience. SCI rehabilitation professionals in the SCI MDT have an exceptional opportunity to provide not only the best physical rehabilitation, but also optimal guidance through improving psychosocial health in their patients, which will continue to benefit them after discharge into the community [5, 6, 9, 13, 19, 21].

Like anyone, individuals with SCI must adapt continuously throughout their lives, and when faced with barriers, they need to adjust to maintain balance across the biopsychosocial domains mentioned above [5]. Rehabilitation will help them adjust to the multiple challenges they face. This introduces the necessity of viewing adjustment through a multifactorial model lens in which adjustment will be affected by multiple factors, including personal, social, environmental and illness specific factors, all of which can interact with the severity of the SCI and change in social roles, as well as life stressors (present and future), with consequent effects on wellbeing [5]. It is accepted that any attempt to describe the complex process of adjustment using a model format will, by definition, simplify the process [5], and it is important to factor this limitation into any SCI adjustment analyses or assessments.

### The spinal cord adjustment model

The Spinal Cord Adjustment Model (SCIAM) views adjustment after SCI as a multi-factorial, non-linear dynamic of adjustment over time [5, 25]. SCIAM contends that adjustment will be affected by multiple layers of factors, including environmental, social, personal, psychological, and medical factors (moderators) with adjustment affected by what we have labelled the “engine room” of adjustment, that is, an appraisal re-appraisal coping response process (mediators). Evidence from our research and independent research supports the multi-factorial interactive assumptions of SCIAM, especially the moderator-mediator process [5, 25, 26]. Detailed descriptions and illustrations of SCIAM are available [5, 25]. SCIAM contends that moderators will influence adjustment outcomes including any pre-existing conditions, health status at the time of the injury, cultural factors, medications, personality, age, cognitive capacity, pain intensity and interference, psychological distress, biological factors, and religious and political factors [5, 25]. Furthermore, moderators are assumed to influence outcomes in an interactive and reciprocal manner [5, 25].

Crucially, SCIAM emphasises the central role that the engine room plays in determining adjustment outcomes [5, 25]. This mediator process involves a person making decisions about what they believe is important and relevant (appraisal-reappraisal), leading to the adoption of coping strategies. Mediators acting within the engine room include perceptual factors like self-efficacy, cognitive distortions such as catastrophizing thinking, self-blame or blaming others, and self-esteem [5, 25]. With the person thinking things through, this “engine room” dynamic will result in the employment of a coping strategy (or no coping strategy), with the success or failure of the coping strategy or lack of action providing feedback to the person, resulting in a secondary appraisal process that may lead to persistence with that coping strategy, or if a perceived failure, adoption of alternative coping strategies. SCIAM argues that this dynamic is always influenced by moderators at any point in time.

### Illustration of the dynamics of adjustment using SCIAM

Two fictional case examples illustrate how SCIAM explains adjustment outcomes drawing on the interactive role of multiple moderators and mediators.

#### Case 1: negative adjustment

**Fig. 1:**
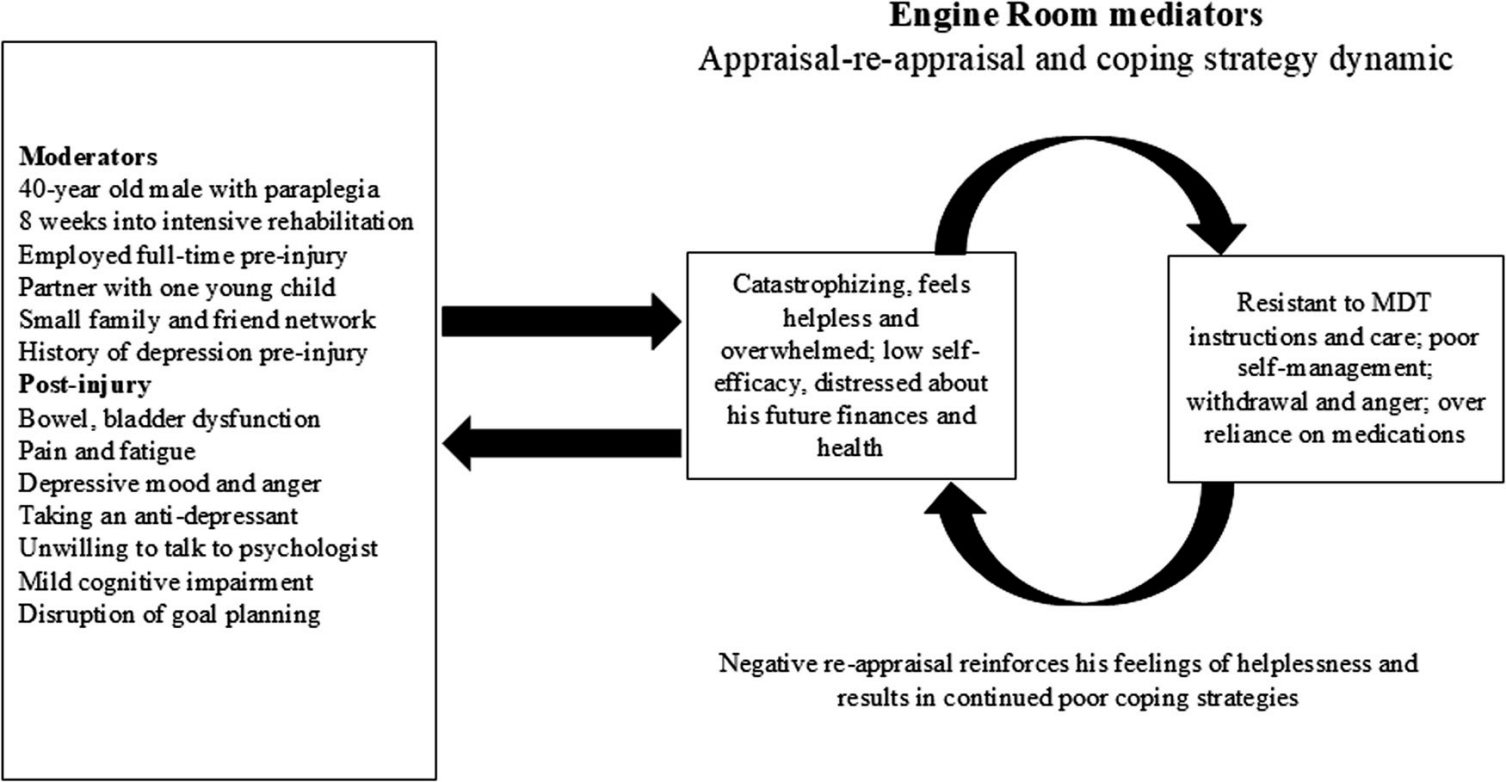
Case example 1 showing negative appraisal and maladaptive adjustment.

Figure 1 shows summary details of a male aged 40-years with an acute traumatic injury and a complete T4-5 paraplegia is 8 weeks into intensive SCI rehabilitation. He was employed full-time before his injury, has a supportive family (wife and one young child) as well as having a small network of family and friends. He is experiencing difficulty with managing his altered bowel and bladder function, which is distressing him. He has a pre-existing history of depression. An assessment reveals that he has clinically elevated depressive mood. A recent cognitive screen suggests he has mild cognitive impairment. He has pain and fatigue. He is taking a serotonin-norepinephrine reuptake inhibitor anti-depressant for his mood and pain. All these moderators act on his appraisal/re-appraisal/coping feedback process (the engine room). He has low self-efficacy, reporting that he is feeling helpless and overwhelmed in his situation. He is catastrophizing about his future health, his ability to support his family, and pain, reinforcing his low self-efficacy. His mild cognitive impairment results in him finding it difficult to focus and comprehend rehabilitation instructions from the MDT. He is feeling angry about his situation and blames external sources like the MDT. He is not interested in talking to the SCI Unit psychologist or social workers about his depression and anger. His low mood interacts with his low self-efficacy and tendency to catastrophize, leading to poor coping strategies, such as resisting instructions and support from the MDT and isolating himself and withdrawing from other patients in the SCI Unit. He is relying very much on his medications to manage his mood and pain. These multiple interactions result in a negative influence on his self-appraisal and adjustment, perhaps delaying his rehabilitation goals and discharge planning. This further negatively impacts psychosocial factors such as his relationships with his partner, family, and friends, increases his distress about his financial position, worsens his mental health, increases pain and confusion, fatigue, and so on. Employing a person-centric multifactorial model, in this case, will assist the MDT to develop applicable goals to counter the barriers to adjustment demonstrated by this patient. Applying SCIAM enables the MDT to take into account the multiple factors related to adjustment, and how these moderators and mediators may interact over time. This will assist the MDT to analyse progress or lack of, and therefore provide direction to counter any barriers to his adjustment. Encouragingly, it may also help mount “engine room” strategies that may boost his self-efficacy and lower catastrophizing thinking.

#### Case 2: positive adjustment

**Fig. 2:**
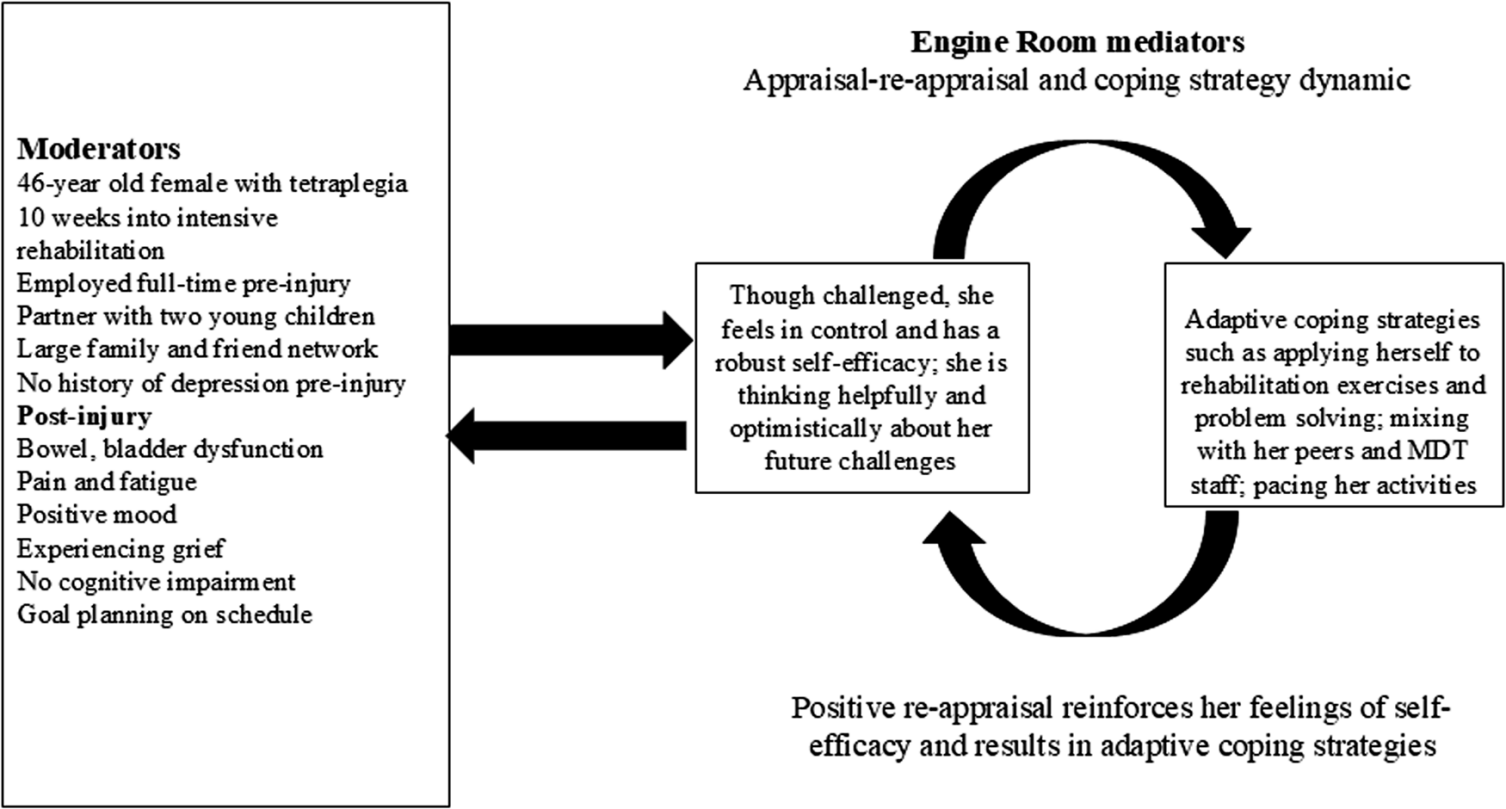
Case example 2 showing positive appraisal and adaptive adjustment.

Figure 2 provides a summary of details for a female aged 46-years who sustained an acute traumatic tetraplegia with a complete C6-7 lesion is 10 weeks into intensive SCI program. She was employed full-time pre-injury, has a supportive partner, two young children, and also has a large network of family and friends. She is experiencing pain and challenges with her bowel and bladder function. She has no pre-existing history of depression. Recent screening reveals her mood is within normal limits though she is experiencing grief about her injury and loss. Cognitive screening suggests her cognitive function is not impaired. She is experiencing fatigue. All these moderators will act on her appraisal/re-appraisal/coping feedback process in the engine room and of course vice versa. Assessment shows she has robust self-efficacy, for example, she reports that though she feels challenged, she believes she has the internal and external resources to manage. While appropriately concerned, she is not catastrophizing about her future health and her ability to support her family, reinforcing her self-efficacy. She also adheres to and understands rehabilitation instructions from the MDT. While there may be strong emotions concerning her SCI and its circumstances, she is trying not to be angry and does not blame herself or others. Her positive mood interacts with her self-efficacy leading to adaptive coping strategies, such as following and contributing to MDT instructions, problem-solving, mixing well in the SCI Unit with her peers and MDT staff, and applying herself diligently to using her rehabilitation skills, such as her physiotherapy and occupational therapy exercises. This is having a positive effect on her rehabilitation goals and discharge planning. Psychosocial factors such as her relationships with her partner, family and friends remains stable and optimistic. Her partner is also actively involved in her rehabilitation journey. Though she is experiencing pain and fatigue, she is open to solutions and external support when required. Employing a person-centric multifactorial model in this case will help the MDT examine how moderators and mediators are interacting, provide direction to strengthen her resilience, develop applicable goals for her ongoing rehabilitation journey, and importantly, with respect to the “engine room” process, help her maintain a robust self-efficacy and adaptive coping process in the face of ongoing challenges she will have to cope with.

### The role of peer support

The role of peer support in the SCI rehabilitation process is central [5]. A peer support person is someone with a lived experience of SCI who can provide practical guidance and support about goals and living with a SCI. The peer support person will also continue to assist the person with SCI, not only during rehabilitation but importantly, after discharge when living in the community. Peer support goals are designed to help the person with an acute SCI to recover optimal independence, strengthen connections within the community, and to offer advice about self-management skills necessary for everyday living (Spinal Cord Injuries Australia; https://scia.org.au/peer-support/). It is likely that those who receive peer mentoring during and after discharge from rehabilitation will experience improvements in psychosocial factors like self-efficacy and resilience [5].

### The need for psychosocial guidelines in SCI rehabilitation

Incorporating psychosocial care guidelines into SCI rehabilitation will I believe, address psychosocial risks and as such, benefit adjustment [6]. However, many MDT professionals working in SCI rehabilitation may not feel confident to apply psychosocial care as part of standard practice [6]. To enhance psychosocial care and PCC within SCI rehabilitation, I firmly believe it advisable to integrate psychosocial guidelines and their recommendations into SCI rehabilitation. We have recently upgraded psychosocial guidelines with recommendations in 2023 and these are available online: https://aci.health.nsw.gov.au/networks/spinal-cord-injury/resources/psychosocial-care.

Given that multiple psychosocial factors (moderators and mediators) influence every aspect of SCI rehabilitation, these 2023 guidelines address the multiple psychosocial domains that can act as facilitators or barriers to adjustment outcomes following SCI [5, 6, 12, 13, 27–30]. An example will help. Healthcare professionals working in the acute and rehabilitation phases need to be confident to screen for symptoms of mild cognitive impairment and mental health comorbidities in their patients, and in response, have the confidence to integrate and adopt psychosocial care strategies that assist in referral processes, as well as promote an environment in which the patient can cope and adjust to the best of their ability. Additionally, the psychosocial guidelines have been designed to support strategies for effective communication between MDT staff and patients, with the aim of being able to create a unified team approach that will support and motivate patients with SCI during their rehabilitation journey. To gain a detailed grasp of the psychosocial guidelines, the reader is advised to consult the psychosocial guidelines mentioned above. It must be clear that the psychosocial guide is not a substitute for the knowledge and skill of individual practitioners, nor does it attempt to impose clinical guidelines on medical and physical treatments.

## Discussion

SCI is a very complex injury with permanent disability and lifelong impacts, so it is critical that MDT professionals understand the considerable influence that psychosocial factors will have on the ability of persons to adjust to SCI [5, 6]. With this in mind, five factors with a psychosocial source were presented and discussed, pointing out how they interact with each other and how they may influence adjustment. Furthermore, it is essential that SCI rehabilitation be person-centred, with the inclusion of psychosocial guidelines being an important consideration for achieving PCC. These challenges have been discussed in the light of an overarching unifying framework called the Biopsychosocial Model. Employing this framework, a multi-factorial model that explains the complex non-linear processes involved in adjustment after SCI was presented, called SCIAM. This model was designed to clarify how numerous factors, namely moderators and mediators, can influence the complex process of adjustment over time. Importantly, the relationship of this adjustment model to the processes involved in SCI rehabilitation were also discussed, with an intention of assisting the MDT to consider how a better understanding of the complexity of adjustment can improve rehabilitation outcomes. Two case studies were provided to illustrate how this framework and SCIAM can be used to address barriers and facilitators to adjustment.

### Clinical implications

There are important clinical implications concerning psychosocial factors and adjustment dynamics. It is my firm belief that SCI adjustment will be enhanced if a multifactorial model approach is used by the MDT to clarify how multiple non-linear psychosocial factors interact with each other and with other non-psychosocial moderators to act as possible barriers or facilitators to adjustment. These may include age, education, personality, traumatic versus non-traumatic, level of injury, completeness of lesion, medications and so on. The model then provides clinical guidance on how to analyse risk and benefit. For example, it provides an “engine room” appraisal-reappraisal and coping process to be considered for possible interventions for the team. Judiciously using this information, the MDT can assess and strategize to reduce the influence of barriers or strengthen facilitators. Finally, employing a PCC approach is paramount, including peer support and an integration of psychosocial guidelines and recommendations for the MDT to assist in setting goals and discharge planning. I hope this invited Guttmann lecture paper stimulates researchers and clinicians to consider the complexity involved in the adjustment process, and to apply these dynamics in clinical aspects of SCI rehabilitation and research.
